# The anesthetic approach for endovascular recanalization therapy depends on the lesion site in acute ischemic stroke

**DOI:** 10.1007/s00234-021-02762-3

**Published:** 2021-07-10

**Authors:** Kilian Fröhlich, Gabriela Siedler, Svenja Stoll, Kosmas Macha, Thomas M. Kinfe, Arnd Doerfler, Felix Eisenhut, Tobias Engelhorn, Philip Hoelter, Stefan Lang, Iris Muehlen, Manuel Schmidt, Bernd Kallmünzer, Stefan Schwab, Frank Seifert, Klemens Winder, Michael Knott

**Affiliations:** 1 Department of Neurology, University Hospital Erlangen, Friedrich-Alexander-University Erlangen-Nürnberg (FAU), Schwabachanlage 6, 91054 Erlangen, Germany; 2 Department of Neurosurgery, University Hospital Erlangen, Friedrich-Alexander-University Erlangen-Nürnberg (FAU), Schwabachanlage 6, 91054 Erlangen, Germany; 3 Department of Neuroradiology, University Hospital Erlangen, Friedrich-Alexander-University Erlangen-Nürnberg (FAU), Schwabachanlage 6, 91054 Erlangen, Germany

**Keywords:** Acute ischemic stroke, Endovascular therapy, Voxel-based lesion symptom mapping, Neuroimaging

## Abstract

**Purpose:**

Endovascular therapy (EVT) of large-vessel occlusion in acute ischemic stroke (AIS) may be performed in general anesthesia (GA) or conscious sedation (CS). We intended to determine the contribution of ischemic cerebral lesion sites on the physician’s decision between GA and CS using voxel-based lesion symptom mapping (VLSM).

**Methods:**

In a prospective local database, we sought patients with documented AIS and EVT. Age, stroke severity, lesion volume, vigilance, and aphasia scores were compared between EVT patients with GA and CS. The ischemic lesions were analyzed on CT or MRI scans and transformed into stereotaxic space. We determined the lesion overlap and assessed whether GA or CS is associated with specific cerebral lesion sites using the voxel-wise Liebermeister test.

**Results:**

One hundred seventy-nine patients with AIS and EVT were included in the analysis. The VLSM analysis yielded associations between GA and ischemic lesions in the left hemispheric middle cerebral artery territory and posterior circulation areas. Stroke severity and lesion volume were significantly higher in the GA group. The prevalence of aphasia and aphasia severity was significantly higher and parameters of vigilance lower in the GA group.

**Conclusions:**

The VLSM analysis showed associations between GA and ischemic lesions in the left hemispheric middle cerebral artery territory and posterior circulation areas including the thalamus that are known to cause neurologic deficits, such as aphasia or compromised vigilance, in AIS-patients with EVT. Our data suggest that higher disability, clinical impairment due to neurological deficits like aphasia, or reduced alertness of affected patients may influence the physician’s decision on using GA in EVT.

## Introduction

Contemporary therapeutic options for vascular recanalization in acute ischemic stroke (AIS) include systemic thrombolysis with alteplase and endovascular therapy (EVT) via catheter-based thrombectomy [1]. Whereas intravenous thrombolysis has been established as the cornerstone of medical treatment for ischemic stroke patients, EVT has evolved as standard technique for large-vessel occlusion of the anterior circulation and basilar thrombosis in the posterior circulation [1–3]. EVT may be performed in general anesthesia (GA) or conscious sedation (CS). Several studies have examined the effects and the benefits of GA vs. CS, but the optimal approach is still a matter of debate [4]. Both methods are considered equally safe, as long as hemodynamic stability is guaranteed and no delay in “door to groin-time” for EVT is produced [4]. The anesthetic method of choice for EVT depends on patients’ condition, preference of the treating physician, as well as on organizational and logistic aspects of the local hospital [2, 4, 5]. CS is often used as an easy first-line strategy when patients are cooperative, whereas GA is preferred in patients with excessive movements, respiratory compromise, or loss of consciousness [4, 6–10]. Neurologic symptoms, e.g., aphasia or compromised vigilance, are related to lesions in specific brain regions [11]. Thus, we hypothesize that ischemic lesion location in the brain might influence the physician’s decision on the anesthetic approach. So far, there is no distinct analysis of the relation of neuroimaging data of ischemic cerebral lesion sites and the preferred anesthetic approach, i.e., GA or CS, in AIS patients treated with EVT.

Therefore, we used voxel-based lesion symptom mapping (VLSM) to assess associations between the anesthetic approach (GA or CS) and the cerebral ischemic lesion location.

## Materials and methods

### Patients

This study was a retrospective analysis of AIS patients seen between 2006 and 2016 at the Department of Neurology of the University Hospital Erlangen of the Friedrich-Alexander-University Erlangen-Nürnberg (Erlangen, Germany). The study based on a prospective stroke registry that was designed until and discontinued after 2016, so no patients were included after its ending. Patients were examined and treated by qualified neurologists and neuroradiologists during their admission and stay in the hospital*.* Patients with acute stroke undergoing intravenous thrombolysis and/or EVT and admitted to our stroke center were entered into a prospective database containing baseline demographic variables, information regarding medical history, as well as parameters on the present stroke. Brain imaging data obtained of patients are stored in computerized databases. The indication for intravenous thrombolysis was based on clinical evaluation and imaging following the recommendations of international treatment guidelines [12]. Patients with large-vessel occlusion (internal carotid artery, proximal segment of middle cerebral artery, and basilar occlusion) were considered for endovascular treatment by experienced neurointerventionalists. The decision between GA and CS was made in an interdisciplinary consensus between the treating neurologist and the interventional neuroradiologist. This approach based on clinical experience and did not follow standardized criteria. For the following analysis, only patients undergoing EVT were selected for the study. For the study registry, ethics approval was obtained by the Ethics Committee of FAU Erlangen-Nürnberg (registration number 377_17Bc). All patients or the legally authorized representatives gave written and informed consent to the use of the data*.*

For the study, we retrospectively included patients of the databases who fulfilled the following criteria: (1) patients with AIS receiving EVT, (2) age of at least 18 years, and (3) available medical reports containing details of the medical history. We excluded patients with the following conditions: (1) MRI or CT sequences of poor quality or not available and (2) other structural diseases than stroke.

The degree of stroke disability was determined using the National Institute of Health (NIHSS) score [13]. To compare the imaging characteristics of patients with GA and with CS in patients with EVT in AIS, we established a cohort with patients with GA and a control group of patients with CS out of the databases.

### CT/MRI examination and lesion mapping

All patients underwent MRI with a multimodal protocol (3 T Magnetom Trio or 1.5 T Magnetom Sonata; Siemens Healthineers AG, Erlangen, Germany) or CT (Sensation 64 or Somatom Definition AS + ; Siemens Healthineers AG, Erlangen, Germany) of the brain with a slice thickness of 5-mm maximum. In patients who received both MRI and CT, the lesion was determined using the MRI scan. Patients who received CT but no MRI were only included when a CT scan was acquired > 24 h after stroke onset, and the infarction was demarcated. Imaging modality was a decision of the treating clinician. Patients not eligible for MRI received CT. Lesions were assessed on anonymized diffusion-weighted MRI sequences or non-enhanced CT scans by two experienced raters (KF and MK). Lesions were manually delineated on imaging scans using MRIcron software [14, 15]. The MRI scan and the lesion shape were transferred into stereotaxic space using the normalization algorithm of SPM12 and the Clinical Toolbox for SPM [16–18]. Finally, using the CT or MR-segment-normalize algorithm of the Clinical Toolbox, the CT or MR images were transformed to the T1 template based on younger individuals with a resampled voxel size of 1 × 1 × 1 mm^3^ [16].

### Statistical analysis

Because EVT in patients with posterior circulation infarcts was almost always performed in GA (93.5%), we conducted two separate imaging analysis steps to isolate the effect of anterior circulation infarcts on the decision of the preferred anesthetic method: one for all patients with anterior and posterior circulation and another for anterior circulation infarcts only. For each of both subgroups, we determined the lesion overlap, i.e., the prevalence of identical lesion sites among all patients with AIS and EVT. Dichotomous overlap values of lesion sites identified in the VLSM analysis were correlated with the dichotomous behavioral variable, that is, whether the patient had received GA or CS using the Liebermeister test with 4000 permutations [19, 20]. To control for multiple comparisons, we applied a family-wise error (FWE) correction of *p* < 0.05. Lesion volumes were calculated using the nonparametric mapping (NPM) software implemented in the MRIcron software package [15]. To determine damaged brain regions, affected voxels were overlaid on the Automated Anatomic Labeling (AAL) atlas. The peak coordinates of the involved regions are presented in the Montreal Neurological Institute (MNI) space.

Demographic and clinical data were tested for normal distribution using the Shapiro–Wilk test and are presented as mean and standard deviation or median and interquartile ranges. Data were compared between GA and CS groups using the Chi-square test or Mann–Whitney U-test, as appropriate. Two-tailed p values were calculated; statistical significance was assumed for *p* < 0.05. For statistical calculations, we used commercially available statistic software (SPSS 20.0; IBM, Armonk, NY).

## Results

### Patient characteristics

Of 1209 individuals with AIS and systemic thrombolysis, 201 patients (16.6%) underwent EVT. Of these 201 stroke patients with EVT, 22 patients did not meet the inclusion criteria; 16 patients had no demarcation of infarction; 6 patients had no imaging, imaging of poor quality, or inappropriate imaging sequences. Thus, 179 stroke patients with EVT fulfilled the inclusion criteria and were eligible for the VLSM analysis. In 99 patients, EVT was performed in GA and in 80 patients in CS. Of the 179 patients with EVT, 148 had anterior circulation, and 31 patients had posterior circulation infarcts. Of the 31 patients with posterior circulation infarcts, 29 patients (93.5%) had EVT in GA.

**Fig. 1:**
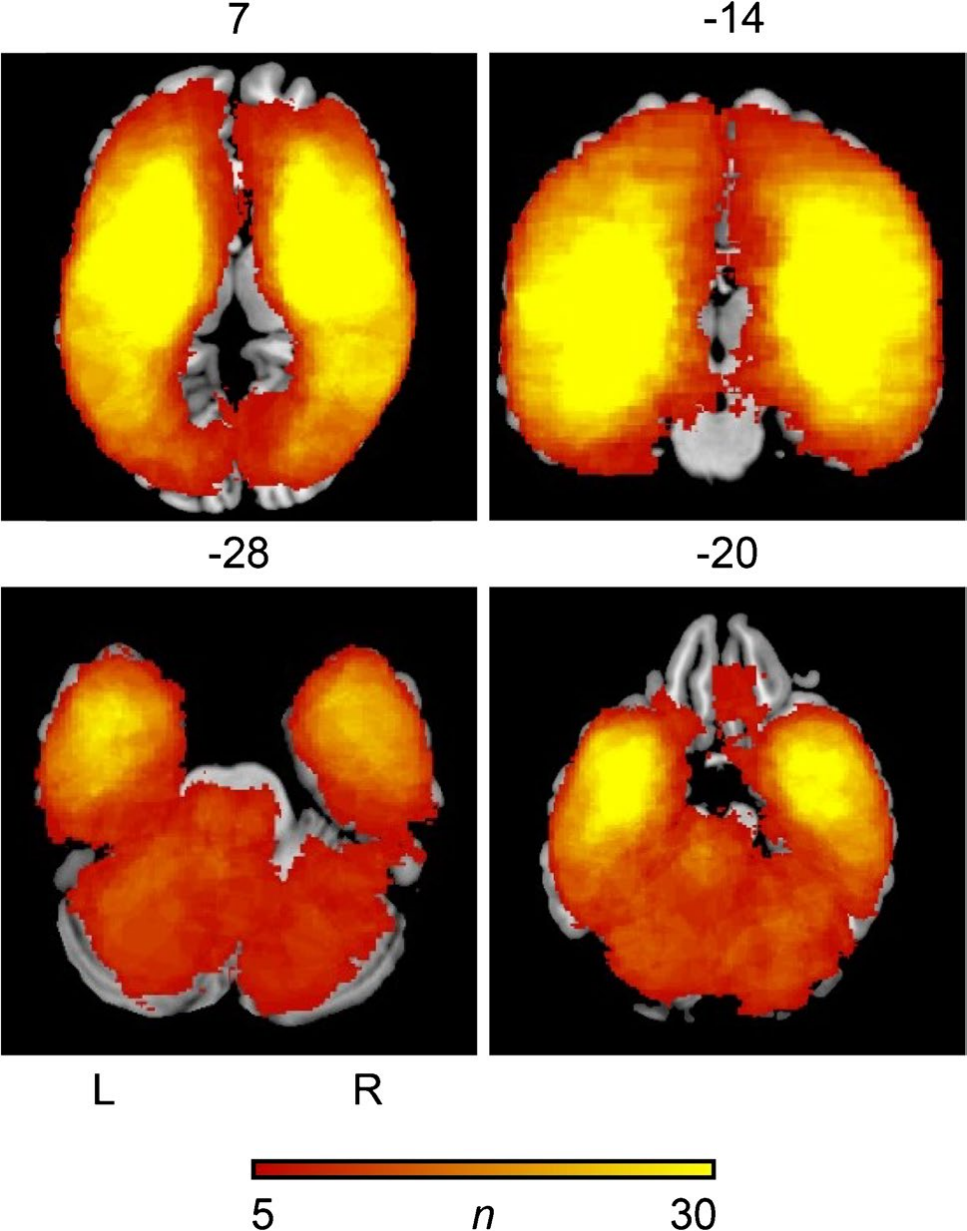
Lesion distribution and lesion overlap of all patients with endovascular therapy with acute ischemic stroke of the anterior and posterior circulation. The number of overlapping lesions is illustrated by different colors coding increasing frequencies from dark red to yellow. The lesion overlap is thresholded to include only voxels that were lesioned in at least 5 individuals. L, left hemisphere; R, right hemisphere; n, number of overlaps with a lesion in a given voxel

The first VLSM analysis included all 179 patients with infarcts of the anterior and posterior circulation. Demographic, clinical parameters and imaging characteristics of stroke patients with GA and CS are demonstrated in Table 1. Glasgow coma scale score and vigilance were significantly lower and the presence of aphasia and aphasia severity significantly higher in the GA group. NIHSS scores and total ischemic lesion volume were significantly higher in the GA compared to the CS group. Age, modified ranking scale (mRS) prior to admission, and rate of systemic thrombolysis with alteplase did not differ between stroke patients with GA and CS. Infarction was more often located in the left hemisphere or in the infratentorial brain regions in the GA group*.* Figure 1 shows the lesion distribution and lesion overlap of all patients with GA and CS patients with EVT and AIS of the anterior and posterior circulation.

**Table 1:** Clinical parameters of all patients with endovascular therapy with acute ischemic stroke of the anterior and posterior circulation

	GA (n = 99)	CS (n = 80)	p
Age (years); median (IQR)	71 (78–58)	74 (82–66)	0.305^a^
NIHSS; median (IQR)	20 (35–16)	15 (19–11)	0.000^a^
Female/male	47/52	42/38	0.504^b^
Lesion volume, voxels; median (IQR)	135,773 (284,926–62,711)	46,535 (160,345–15,900)	0.001^a^
Localization of infarction (left hemispheric/right hemispheric/infratentorial)	42/28/29	36/42/2	0.000^b^
Aphasia type (no deficit/expressive/sensory/global/n.a.)	25/2/2/36/34	47/10/0/23/0	0.000^b^
Aphasia severity (no deficit/mild/medium/severe/n.a.)	25/1/9/31/33	47/12/8/13/0	0.000^b^
Glasgow coma scale (IQR; 0–15 points)	10 (15–7)	15 (15–13)	0.029^a^
Vigilance (awake/somnolence/sopor/coma/n.a.)	29/28/13/5/24	67/13/0/0/0	0.000^b^
Pre-mRS (0/1/2/3/4)	52/14/19/9/2	50/10/6/9/3	0.209^b^
Thrombolysis (yes/no)	79/20	66/14	0.647^b^

*CS* conscious sedation, *GA* general anesthesia, *IQR* interquartile range, *n.a.* data not available, *NIHSS* National Institute of Health Stroke Scale, *mRS* modified ranking scale

^a^p Value derived from Mann–Whitney U-test

^b^p Value derived from Chi-square test

**Fig. 2:**
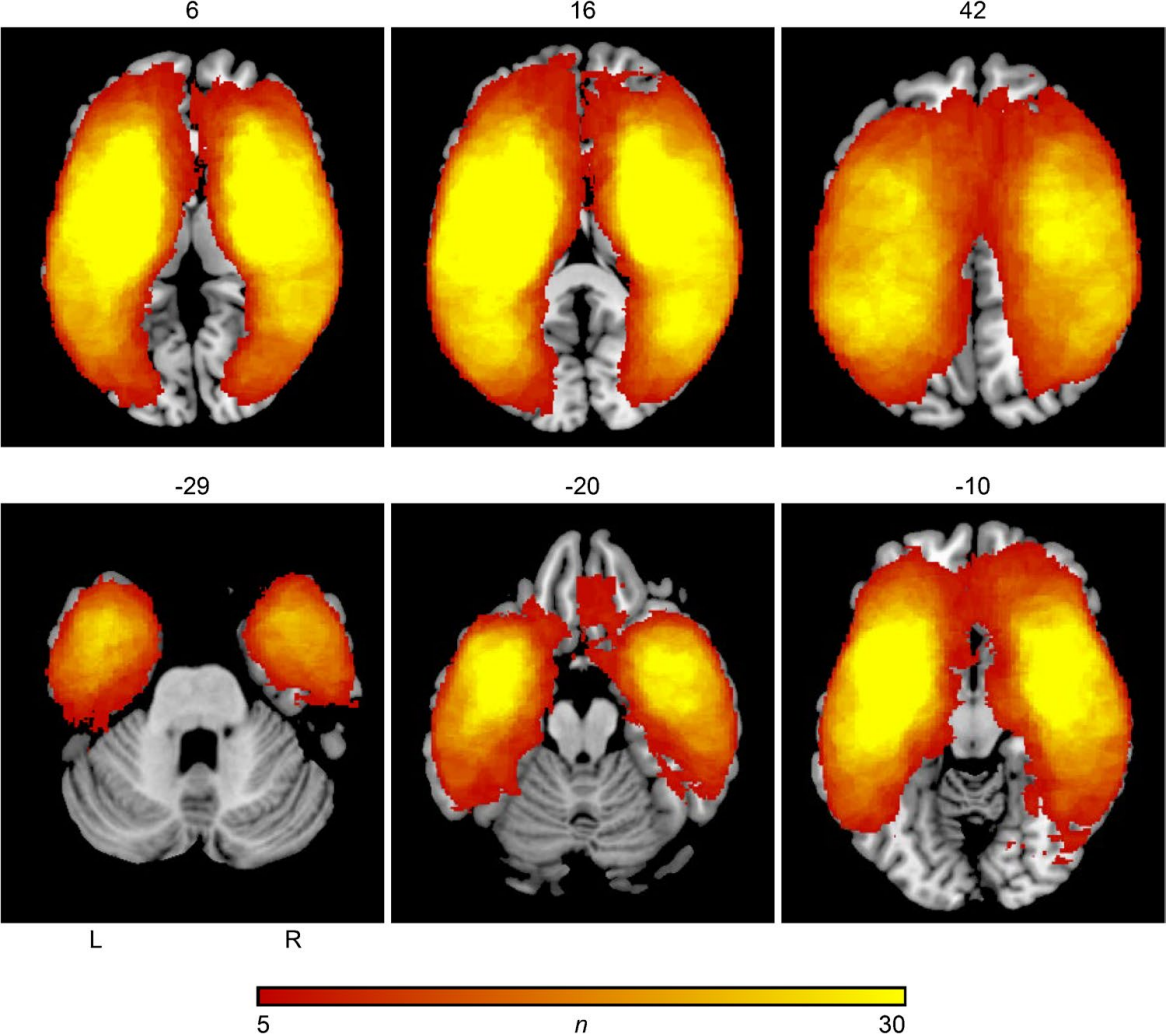
Lesion distribution and lesion overlap of all patients with endovascular therapy with acute ischemic stroke of the anterior circulation only. The number of overlapping lesions is illustrated by different colors coding increasing frequencies from dark red to yellow. The lesion overlap is thresholded to include only voxels that were lesioned in at least 5 individuals. L, left hemisphere; R, right hemisphere; n, number of overlaps with a lesion in a given voxel

The second VLSM analysis included only the 148 patients with infarcts of the anterior circulation. Demographic, clinical parameters and imaging characteristics of patients with anterior circulation strokes are demonstrated in Table 2. Levels of vigilance were significantly lower in GA patients. Aphasia was more frequent and more severe in the GA group. NIHSS scores and total lesion volume were significantly higher in the GA group compared to CS*.* Age, modified ranking scale (mRS) prior to admission, rate of systemic thrombolysis with alteplase, and Glasgow coma scales scores did not differ between both groups. Localization of infarction territory did not differ between both groups. Figure 2 shows the lesion distribution and lesion overlap of all patients with GA and CS in the subgroup of patients with EVT and AIS of the anterior circulation only.

**Table 2:** Clinical parameters of all patients with endovascular therapy with acute ischemic stroke of the anterior circulation only

	GA (n = 70)	CS (n = 78)	p
Age (years); median (IQR)	72 (78–58)	74 (82–66)	0.390^a^
NIHSS; median (IQR)	20 (23–16)	15 (19–11)	0.000^a^
Female/male	33/37	41/37	0.510^b^
Lesion volume, voxels; median (IQR)	117,037 (319,647–87,988)	46,535 (150,431–16,338)	0.000^a^
Localization of infarction (left hemispheric/right hemispheric)	42/28	36/42	0.092^b^
Aphasia type (no deficit/expressive/sensory/global/n.a.)	15/2/2/35/16	46/9/0/23/0	0.000^b^
Aphasia severity (no deficit/mild/medium/severe/n.a.)	15/1/9/30/15	46/11/8/13/0	0.000^b^
Glasgow coma scale (IQR; 0–15 points)	12 (15–9)	15 (15–13)	0.161^a^
Vigilance (awake/somnolence/sopor/coma/n.a.)	24/24/9/1/12	66/12/0/0/0	0.000^b^
Pre-mRS (0/1/2/3/4)	37/9/13/6/2	48/10/6/9/3	0.366^b^
Thrombolysis (yes/no)	57/13	65/13	0.761^b^

*CS* conscious sedation, *GA* general anesthesia, *IQR* interquartile range, *n.a.* data not available, *NIHSS* National Institute of Health Stroke Scale, *mRS* modified ranking scale

^a^p Value derived from Mann–Whitney U-test

^b^p Value derived from Chi-square test

### Voxel-based lesion symptom mapping

**Fig. 3:**
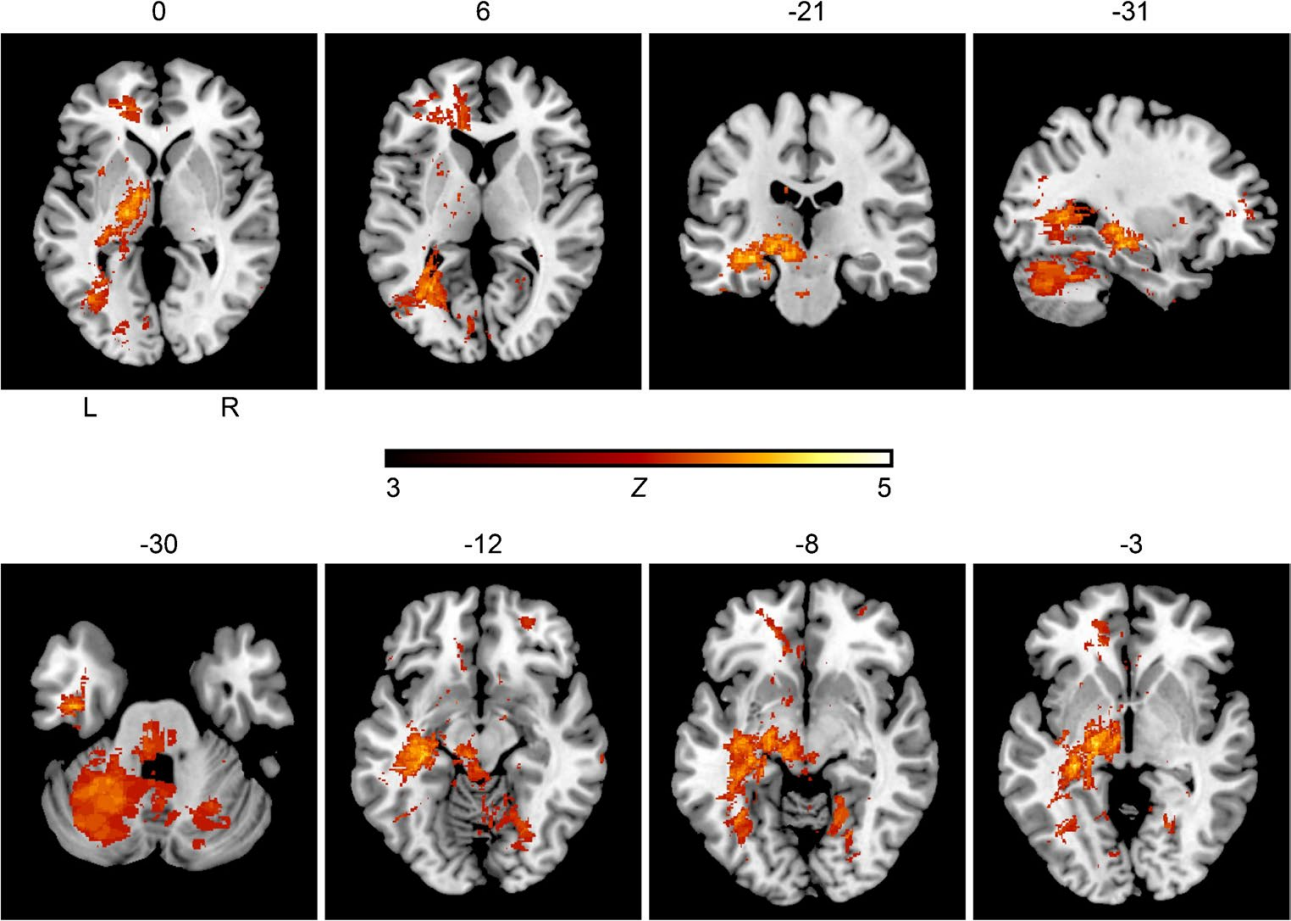
Results of the voxel-wise lesion-symptom mapping analysis of patients receiving endovascular therapy with acute ischemic stroke of the anterior and posterior circulation. We conducted the nonparametric Liebermeister statistics with 4000 permutations to assess correlations. Lesioned voxels in the posterior circulation including the pons, cerebellum and thalamus, and the left temporal lobe were most prominently associated with general anesthesia as the preferred anesthetic approach in acute ischemic stroke patients receiving endovascular therapy. A family wise error (FWE) correction of q < 0.05 was applied (z-score = 3.0). L = left hemisphere; R = right hemisphere; z = z-score

The first VLSM analysis including all ischemic infarcts, i.e., anterior and posterior circulation, showed associations between GA as the preferred anesthetic method and ischemic stroke lesion location in a total of 54,671 voxels. Table 3 illustrates affected brain areas of the anterior and posterior circulation associated with GA according to the Automated Anatomical Labeling (AAL) atlas, the corresponding voxel count, and the peak coordinates in MNI space. Figure 3 demonstrates the results of the Liebermeister test of AIS of the anterior and posterior circulation. GA in patients with AIS of the anterior and posterior circulation treated with EVT correlated significantly with brain regions mainly located in the left thalamus, basal ganglia, the left temporal lobe, and brain regions of the posterior circulation.

**Table 3:** Results from the voxel-wise Liebermeister analysis of acute ischemic stroke of the anterior and posterior circulation

Lesion site	Voxels	x	y	z
Frontal sup	214	-28	47	1
Frontal mid (l)	475	-29	49	4
Frontal mid orb (r)	250	27	43	-14
Frontal inf tri (l)	146	-38	39	6
Frontal sup media (l)	733	-17	50	20
Frontal med orb (l)	170	-11	29	-11
Rectus (r)	122	11	16	-15
Cingulum ant (l)	1094	-14	44	9
Calcarine (l)	1079	-27	-64	8
Lingual (l)	406	-13	-59	5
Lingual (r)	901	22	-70	-9
Occipital mid (l)	1444	-40	-65	2
Occipital inf (l)	349	-38	-59	-8
Fusiform (l)	2045	-35	-29	-14
Fusiform (r)	648	25	-66	-12
Putamen (l)	234	-35	-14	-7
Pallidum (l)	179	-21	-6	-4
Thalamus (l)	1555	-19	-16	0
Temporal mid (l)	211	-45	-2	-24
Temporal inf (l)	2211	-42	-13	-31
Cerebellum (l)	10,226	-29	-50	-30
Cerebellum (r)	5035	37	-58	-23
Vermis	1464	-1	-34	-13
Pons	699	-7	-33	-32
Corpus callosum	720	-14	33	5
Internal capsule (l)	906	-18	-18	-3
Ant corona radiata (l)	561	-18	36	12
Post thalamic radiation (l)	1185	-33	-60	4

Brain areas according the areas defined in the Automated Anatomical Labeling (AAL) atlas in which 54,671 lesioned voxels were associated with intubation, as well as corresponding voxel counts, and peak coordinates in MNI space are shown (brainstem and subcortical lesions not included). Only areas with at least 100 lesioned voxels are demonstrated. *l* left, *r* right

**Fig. 4:**
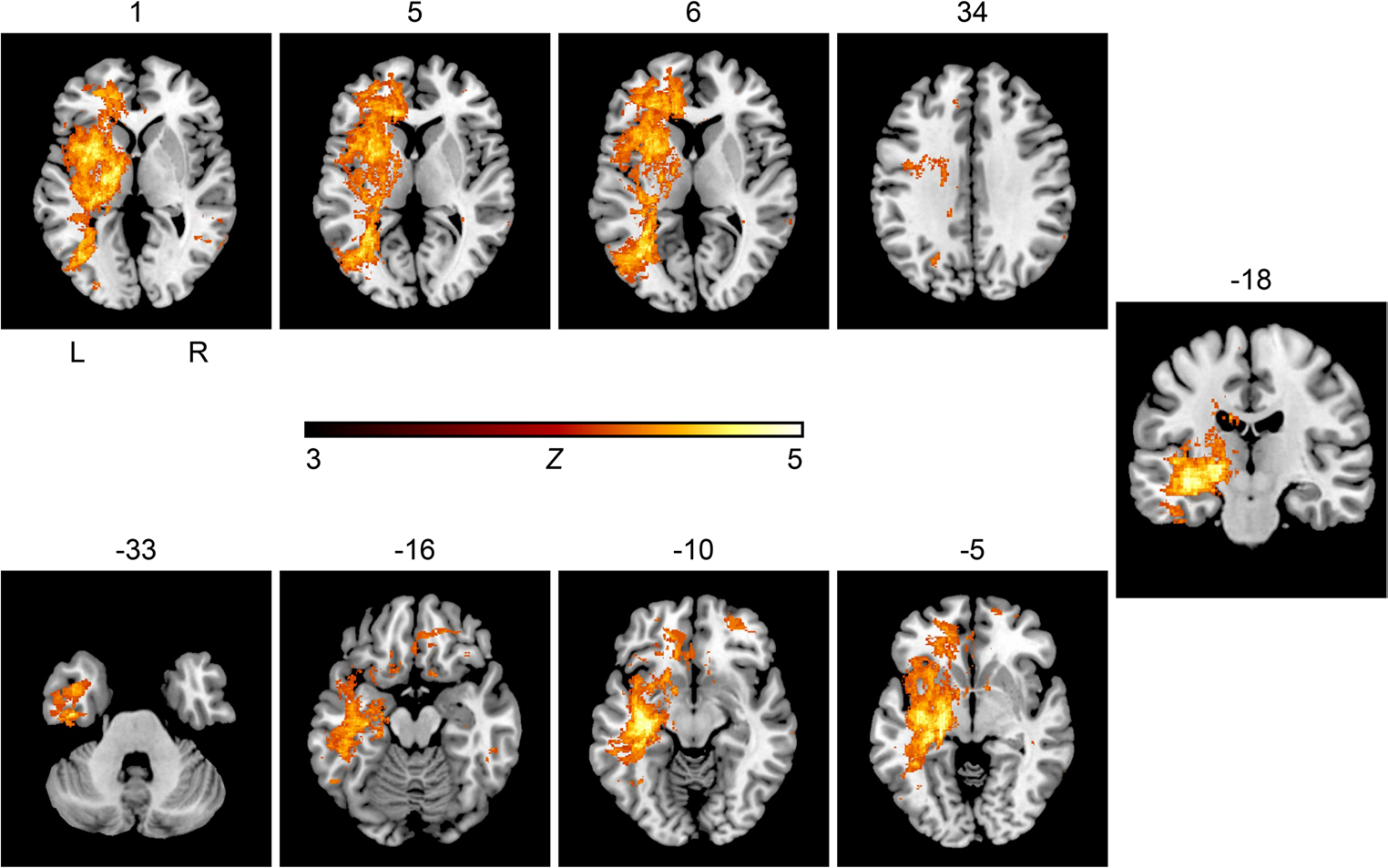
Results of the voxel-wise lesion-symptom mapping analysis of patients receiving endovascular therapy with acute ischemic stroke of the anterior circulation only. We conducted the nonparametric Liebermeister statistics with 4000 permutations to assess correlations. Lesioned voxels in the left middle cerebral artery territory, especially the basal ganglia and the left temporal lobe, were most prominently associated with general anesthesia as the preferred anesthetic approach in acute ischemic stroke patients receiving endovascular therapy. A family wise error (FWE) correction of q < 0.05 was applied (z-score = 3.0). L = left hemisphere; R = right hemisphere; z = z-score

In the second VLSM analysis of anterior circulation infarcts only, the nonparametric voxel-wise analysis using Liebermeister statistics showed associations between AIS and GA in a total of 104,214 lesioned voxels. Table 4 lists the brain areas with the lesioned voxels resulting from the Liebermeister analysis of AIS of the anterior circulation only according to the AAL atlas, the corresponding voxel count, and the peak coordinates in MNI space. Figure 4 demonstrates the results of the Liebermeister test of AIS of the anterior circulation. GA in patients with AIS of the anterior circulation treated with EVT correlated significantly with brain regions of the left hemisphere, the thalamus, basal ganglia, and the temporal lobe.

**Table 4:** Results from the voxel-wise Liebermeister analysis of patients with acute ischemic stroke of the anterior circulation only

Lesion site	Voxels	x	y	Z
Frontal sup (l)	1105	-28	47	1
Frontal mid (l)	2008	-29	49	4
Frontal inf oper (l)	1347	-48	9	9
Frontal mid orb (r)	557	27	43	-14
Frontal inf tri (l)	2834	-42	31	15
Frontal inf orb (l)	1124	-34	25	-7
Rolandic oper (l)	1599	-34	6	15
Frontal sup medial (l)	2547	-13	47	7
Frontal med orb (l)	597	-11	29	-11
Rectus (r)	557	11	16	-15
Insula (l)	8602	-36	-13	-3
Cingulum ant (l)	2197	-14	44	9
Cingulum ant (r)	706	5	31	-3
Hippocampus (l)	4496	-31	-30	-3
Parahippocampal (l)	539	-24	-24	-16
Amygdala (l)	556	-22	0	-13
Occipital mid (l)	4471	-40	-65	2
Fusiform (l)	1325	-35	-29	-14
Caudate (l)	1560	-19	19	9
Putamen (l)	5645	-35	-14	7
Pallidum (l)	1859	-21	-6	-4
Thalamus (l)	2842	-19	-16	0
Temporal sup (l)	1483	-40	-8	-10
Temporal pole sup (l)	1336	-35	9	-29
Temporal mid (l)	3443	-45	-2	-24
Temporal inf (l)	5361	-42	-13	-31

Brain areas according the areas defined in the Automated Anatomical Labeling (AAL) atlas in which 104,214 lesioned voxels were associated with general anesthesia, as well as corresponding voxel counts, and peak coordinates in MNI space are shown (infratentorial, brainstem and subcortical lesions not included). Only areas with at least 500 lesioned voxels are demonstrated. *l* left, *r* right

## Discussion

The VLSM analysis showed associations between GA as the preferred anesthetic method and left hemispheric as well as posterior circulation ischemic infarcts in AIS patients treated with EVT. Left hemispheric lesion clusters were located in the middle cerebral artery territory and included mainly the basal ganglia and temporal lobe (Figs. 3 and 4). In addition, stroke patients with EVT and GA had significantly higher stroke severity, lesion volumes, aphasia scores, and more compromised vigilance scores compared to stroke patients with CS (Tables 1 and 2).

Neurological deficits are related to lesions of distinct brain areas [21, 22]. The influence of neurological deficits and cerebral ischemic lesion location on the decision of GA vs. CS for EVT in AIS has not been studied yet. Therefore, in the current study, we applied a voxel-wise lesion mapping approach to identify lesion clusters in the brain associated with GA or CS. The advantage of VLSM is that it does not predefine lesion sites that assumed to be associated with clinical variables but has the advantage of determining associations between the voxel-wise lesion overlap in any brain area [19].

As a first major finding, the VLSM analysis yielded associations between GA and lesions of the posterior circulation, notably the cerebellum, thalamus, and the pons. In strokes of the posterior circulation, especially basilar occlusion, patients are often compromised in respiratory function, state of vigilance, and cooperation due to acute brainstem disorder or thalamic involvement [3, 23, 24]. Therefore, EVT in patients with strokes in the posterior circulation is mostly performed in GA [3, 23, 24]. In accordance, in our study, 93.5% of patients with EVT due to posterior circulation infarcts had GA. Moreover, our patients with GA had significantly lower levels of vigilance. Although data comparing the use of GA and CS in acute stroke of the posterior circulation is scarce, stroke severity and compromised vigilance may be responsible for the use of GA in posterior circulation strokes [23, 24]. Patient condition is an essential factor for the choice of anesthetic strategy during EVT [4]. While GA constitutes the preferred anesthetic method in stroke patients with higher disability, unstable vital parameters, hypoventilation, loss of consciousness, and excessive movements, CS is often conducted in cooperative patients and is sometimes the preferred first-line strategy to avoid a delay of EVT [6–10, 25, 26].

Moreover, our analysis of infarcts of the anterior circulation showed associations between GA and large lesion clusters in the left basal ganglia, the cerebral white matter including the corticospinal tract, and the left temporal lobe. Lesions of the capsula interna and the corticospinal tract result in a contralateral hemiparesis [27].

Neurologic deficits, e.g., the presence of aphasia, might also influence the decision of the preferred anesthetic method, i.e., GA or CS, since they have a significant impact on stroke severity and patients’ compliance [2, 4, 5, 8, 27].

Our study revealed a lateralization of anterior circulation ischemic infarcts to the left hemisphere in patients with GA. The left hemisphere is the dominant hemisphere for language production and comprehension in the majority of right-handed people [28, 29]. Lesions of the left temporal lobe may result in speech impairment, especially aphasia, and consecutively impaired compliance [22]. In our study, stroke patients who underwent GA had significantly higher aphasia severity scores and were more often affected by aphasia. More precisely, our VLSM analysis revealed that temporal lesions associated with GA were located in the left temporal subcortex of the middle temporal gyrus (MTG) including the hippocampal region, areas contributing to speech recognition and lexical storage [28, 30]. The MTG seems to play a major role in the development of speech comprehension deficits of Wernicke’s aphasia [22]. The results indicate that speech deficits like sensory aphasia that compromise the cooperation of patients may influence the physician’s decision for GA in patients with AIS needing EVT.

### Limitations

Several limitations of this study have to be considered in the interpretation of the results. Although we were able to recruit a large cohort of individuals, a higher sample size may have produced clearer results. However, for a VLSM study, the analysis was adequately powered [20]. Performing a multivariate regression analysis with lesion volume or the level of vigilance as regressors in the Liebermeister test did not produce significant results. After 2016 and the end of our study, EVT became treatment of choice, and major advances in thrombectomy have been made. Although we cannot rule out this may have influenced our results, we do not think that these technological advances have a major impact on the decision on the anesthetic approach and effect on the statistical VLSM comparison of cerebral lesion patterns between the GA and CS groups. Physicians were not questioned about the reasons for their decision between GA and CS, which would have been a valuable additional information. We used imaging protocols with both CT and MRI scans, which could have affected the analysis. Unfortunately, CT perfusion imaging is not validated for the VLSM analysis, so we used CT scans > 24 h after admission to ensure that the infarction was well demarcated. However, using this approach that has been applied in previous studies, “tissue at risk” that contributed to the clinical symptoms prior to EVT may not be included [21, 31]. The sole use of MRI scans would have been preferable, as tissue damage in stroke may be diffuse and not detectable in CT, but also play a role in the development of neurologic deficits [32].

## Conclusions

In conclusion, we postulate that the clinical presentation and severity of symptoms due to the location of lesions may be crucial for physicians to favor GA over CS in EVT of AIS. The analysis revealed lesions of the middle cerebral artery territory, basal ganglia, the left temporal lobe, and of brain regions of the posterior cerebral circulation, especially the left thalamus, to be associated with GA. Additionally, patients with GA were more often affected by aphasia and had higher aphasia severity and lower vigilance scores. Following our results, the higher disability, poorer clinical presentation, and lower cooperation due to aphasia of patients with those lesion locations may influence the treating physicians to favor GA in EVT treatment.

## Data Availability

Authors will review requests for access to the data that support the findings of this study, and access will be granted upon reasonable request.
